# CLARITY: a Shiny app for interactive visualisation of the bovine physical-genetic map

**DOI:** 10.3389/fgene.2023.1082782

**Published:** 2023-05-30

**Authors:** Nina Melzer, Saber Qanbari, Xi Ding, Dörte Wittenburg

**Affiliations:** Research Institute for Farm Animal Biology (FBN), Dummerstorf, Germany

**Keywords:** single nucleotide polymorphism, linkage, recombination rate, education, mapping function

## Abstract

The arrangement of markers on the genome can be defined in either physical or linkage terms. While a physical map represents the inter-marker distances in base pairs, a genetic (or linkage) map pictures the recombination rate between pairs of markers. High-resolution genetic maps are key elements for genomic research, such as fine-mapping of quantitative trait loci, but they are also needed for creating and updating chromosome-level assemblies of whole-genome sequences. Based on published results on a large pedigree of German Holstein cattle and newly obtained results with German/Austrian Fleckvieh cattle, we aim at providing a platform that allows users to interactively explore the bovine genetic and physical map. We developed the R Shiny app CLARITY available online at https://nmelzer.shinyapps.io/clarity and as R package at https://github.com/nmelzer/CLARITY that provides access to the genetic maps built on the Illumina Bovine SNP50 genotyping array with markers ordered according to the physical coordinates of the most recent bovine genome assembly ARS-UCD1.2. The user is able to interconnect the physical and genetic map for a whole chromosome or a specific chromosomal region and can inspect a landscape of recombination hotspots. Moreover, the user can investigate which of the frequently used genetic-map functions locally fits best. We further provide auxiliary information about markers being putatively misplaced in the ARS-UCD1.2 release. The corresponding output tables and figures can be downloaded in various formats. By ongoing data integration from different breeds, the app also facilitates comparison of different genome features, providing a valuable tool for education and research purposes.

## 1 Introduction

Genomic research involving gene mapping of economically important traits, population-specific genetic structure and evolutionary history relies heavily on genetic maps built on the extent of linkage disequilibrium (LD) between genomic markers (e.g., Georges et al., 2019; Johnsson and Jungnickel, 2021). For example, to what extent LD persists in a certain genomic region determines the number of markers required to fine-map a quantitative trait loci with succinct power and precision (for review see Qanbari, 2020). Moreover, genetic maps are valuable resources for comparative genomic analyses among breeds or species (e.g., Everts-van der Wind et al., 2005; Womack, 2005). Of utmost topical importance, however, is the contribution of genetic maps (also known as linkage maps) to measuring haplotype similarity in the context of genomic selection (Musa, 2021) and to chromosome-level assemblies of whole-genome sequences (e.g., De los Ríos-Pérez et al., 2020; Rosen et al., 2020).

Given the value of cattle in sustaining the world food security, the bovine genome is subject of vast amount of ongoing research. We recently updated the genetic map of German Holstein breed (Qanbari and Wittenburg, 2020) and compared it with physical coordinates of the most recent bovine reference genome assembly ARS-UCD1.2 (Rosen et al., 2020). As an extension to this resource, here we introduce a Shiny app CLARITY which facilitates interactive visualisation of the bovine genetic and physical map. CLARITY illustrates the details of male recombination across the bovine genome of selected breeds and suggests suitable genetic-map functions. In addition to published findings, results have been updated by taking most recent knowledge about putatively misplaced markers in the bovine genome assembly into account (Qanbari et al., 2022). Moreover, a linkage map for German/Austrian Fleckvieh cattle has been created. The CLARITY app can therefore serve as a toolkit for both educational and research purposes for the genome of bovine and related species.

## 2 Data preparation

**FIGURE 1:**
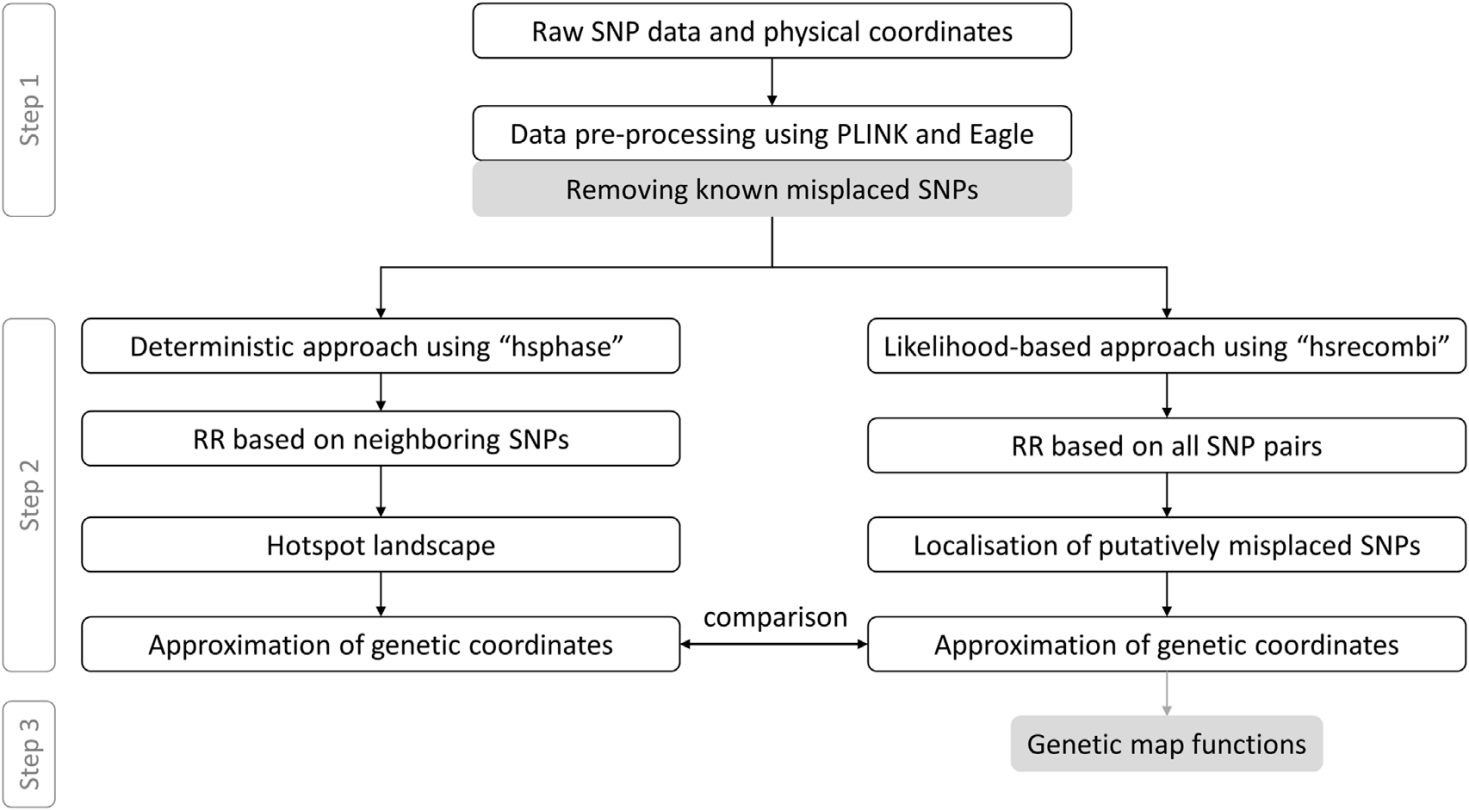
Data processing from raw genotypes to genetic-map functions. This workflow with grey coloured boxes excluded follows Qanbari and Wittenburg (2020) RR, recombination rate.

The app provides access to linkage maps built based on the 50K genotypes of a large pedigree of German Holstein cattle (1,053 half-sib families which comprise 367,056 genotyped animals) as well as German/Austrian Fleckvieh cattle (298,850 genotyped animals pedigreed across 6,866 half-sib families) and based on estimates of recombination rate between intra-chromosomal marker pairs. In what follows, we briefly describe the workflow towards genetic coordinates as depicted in Figure 1 according to Qanbari and Wittenburg (2020). As some of the steps require several days of computing or visual inspection, the workflow was executed once in advance; the app itself dynamically processes physical and genetic coordinates as well as pairwise recombination rates. If not stated otherwise, data processing was executed in R v4.1.3 (R Core Team, 2022).

Step 1: Genotype data were filtered for minor allele frequency >1% and for Mendelian inconsistencies both on marker and individual level using PLINK v1.9 (Purcell et al., 2007) with recommended settings. Genotypes with a Mendelian inheritance error were set to “NA” and missing values were imputed using Eagle v2.4.1 program (Loh et al., 2016). Putatively misplaced markers, which have recently been reported (Qanbari et al., 2022), were discarded. Data passed on to Step 2 comprised 876 half-sib families with 366,565 progeny genotyped at 44,696 single nucleotide polymorphisms (SNPs) in Holstein and 1,577 half-sib families with 270,636 progeny genotyped at 40,003 SNPs in Fleckvieh.

Step 2: Paternal recombination rates to build the genetic map were derived from two different methods, see Figure 1. First, the *deterministic approach* developed by Ferdosi et al. (2014), which is implemented in the R package hsphase v2.0.2, yielded estimates of recombination rate between adjacent markers. These estimates were later used to form the landscape of recombination hotspots. Genetic coordinates were estimated as cumulative sum of recombination rates between neighbouring markers. Second, the *likelihood-based approach*, as implemented in the R package hsrecombi v0.3.4 (Wittenburg, 2020), was applied to estimate recombination rates between all intra-chromosomal marker pairs. These estimates of recombination rate also enabled the identification of candidate misplaced markers in the current assembly release ARS-UCD1.2 (Qanbari and Wittenburg, 2020). Genetic coordinates were obtained with a smoothing approach, in which all recombination rates < 0.05 between any intra-chromosomal marker pairs were taken into account.

Step 3: As a novel contribution, the relationship between recombination rate and genetic distance between all intra-chromosomal marker pairs was investigated for a set of commonly used genetic-map functions (Haldane, 1919; Rao et al., 1977; Felsenstein, 1979; Liberman and Karlin, 1984). Given the estimates of recombination rate ${\hat{\theta }}_{i,j}$ between two markers *i* and *j* and its genetic distances $d_{i,j}$ derived in Step 2 (Qanbari and Wittenburg, 2020), a genetic-map function $f\left(d_{i,j}|a\right)=\theta_{i,j}$ was fitted to the data by solving the following minimisation problem in terms of the model parameter *a* (where *p* is the total number of markers per chromosome):

$$\sum_{\begin{array}{l} i,j=1\\ i<j \end{array}}^{p}{\left({\hat{\theta }}_{i,j}-f\left(d_{i,j}|a\right)\right)}^{2}\to \min\;s.t.\;a\in R$$

We solved this optimisation problem using the R function optim with “Brent” option allowing to specify restrictions on *a*. Rao’s system of mapping function requires $a\in \left(0,1\right)$ . Furthermore, instead of Haldane’s original map function, we investigated a scaled version thereof, i.e.

$$f\left(d_{i,j}|a\right)=\frac{1}{2}\left(1-e^{-2ad_{i,j}}\right)\;with\;a>0$$

For the Binomial map function of Liberman and Karlin (1984), we employed a grid search over $a\in \left\{2,3,4,5\right\}$ seeking the minimum squared deviation as described above. The fitted function leading to the least squared deviation constituted the “best” genetic-map function.

## 3 Implementation

The CLARITY app is an R Shiny web GUI for various operating systems. It relies on several R packages to enable the outcome and visualisation functionalities. CLARITY was implemented in R v4.1.3 (R Core Team, 2022) with help of the R packages shiny v1.7.1 (Chang et al., 2021) and shinydashboard v0.7.2 (Chang and Borges Ribeiro, 2021) to create a dashboard. The graphical output was produced using the R packages ggplot2 v3.3.5 (Wickham, 2016), plotly v4.10.0 (Sievert, 2020) and ggVennDiagram v1.2.0 (Gao, 2021). To provide additional helpful features for the app, such as hiding or toogle of elements, the R packages shinyjs v2.1.0 (Attali, 2021) and shinycssloaders v1.0.0 (Sali and Attali, 2020) were incorporated. Tables were generated using the R package DT v0.22 (Xie et al., 2022). Further R packages were employed: cachem v1.0.6 (Chang, 2021), config v0.3.1 (Allaire, 2020), dplyr v1.0.10 (Wickham et al., 2022), gridExtra v2.3 (Auguie, 2017), htmltools v0.5.2 (Cheng et al., 2021), magrittr v2.0.3 (Bache and Wickham, 2022), metathis v1.1.2 (Aden-Buie, 2022), rlang v1.0.6 (Henry and Wickham, 2022a), sf v1.0.8 (Pebesma, 2018), RVenn v1.1.0 (Akyol, 2019) and purrr v0.3.5 (Henry and Wickham, 2022b). Eventually, an R package was built from the CLARITY app with use of the R package golem v0.3.2 (Fay et al., 2022), which offers default R files for creating the package as well as for deploying the app. The R package roxygen2 v7.1.2 (Wickham et al., 2021) was employed for package documentation. The processed data (i.e., recombination rates, genetic coordinates and parameters of genetic-map functions) were included as Rdata files in the folder “extdata”.

We optimized the app following recommendations for best practice with lighthouse (Google LLC, 2022). Figures were compressed with the tool Squoosh (Google Chrome Team, 2022), and caching of those figures requiring longer loading was enabled.

The structure of the app relies on modules, which eases a clear and concise organisation. The use of modules and corresponding interfaces to the main shiny ui and shiny server enables a straightforward maintenance of the software. Furthermore, since each module is an independent app with its own interface and server (Di Filippo et al., 2019), future modification and extension of the app are supported.

## 4 Realisation and features

The app has three sidebar menus: “Information”, “Breed analysis” and “Breed comparison” which are described in the following.

### 4.1 Information

**FIGURE 2:**
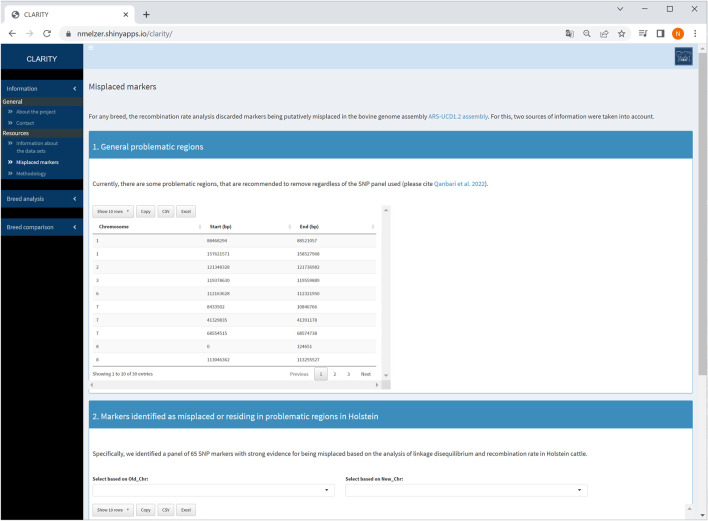
Screenshot of information about problematic regions and list of misplaced markers detected in Holstein cattle.

The first sidebar menu comprises two subitems: (1) general information about the project and contact options as well as (2) details of resources used. More specifically, subitem (2) contains a brief data description for each breed and outlines the methodology used for data analysis and parameter estimation. This subitem also provides auxiliary information about candidate markers identified as being putatively misplaced and/or residing in problematic regions of the ARS-UCD1.2 release (Qanbari et al., 2022, see also Figure 2). These markers were recommended to be excluded from subsequent genomic analyses, such as phasing, imputation or genome-wide association studies.

### 4.2 Breed analysis

Under the second sidebar menu “Breed analysis”, all tabulated and graphical outcomes are presented for the available breeds. So far, options “Holstein” and “Fleckvieh” are available. The user can select a single chromosome or all chromosomes to interconnect the physical and linkage map. The results are divided into different tabs (implemented as separate modules) within the main panel: general information, genetic map, hotspot detection and genetic-map functions. Generally, outputs including tables and figures for a certain interval or for the entire data can also be downloaded for being locally stored. The properties of each tab are explained in more detail below.

#### 4.2.1 General information

**FIGURE 3:**
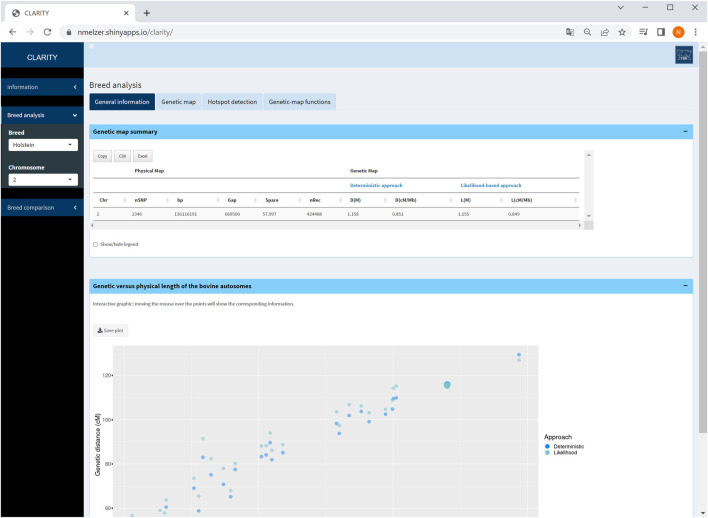
Screenshot of general information on selected chromosome 2. The graphic displays the relationship between genetic and physical map length of all autosomes in Holstein cattle and highlights the selected chromosome.

For all chromosomes, summary statistics are provided about the number of markers considered, total number of recombination events, length of physical and both genetic maps (from Step 2) in tabulated format. This table reduces if a single chromosome is selected (Figure 3). An interactive graphical output displays physical *versus* genetic length per chromosome.

#### 4.2.2 Genetic map

**FIGURE 4:**
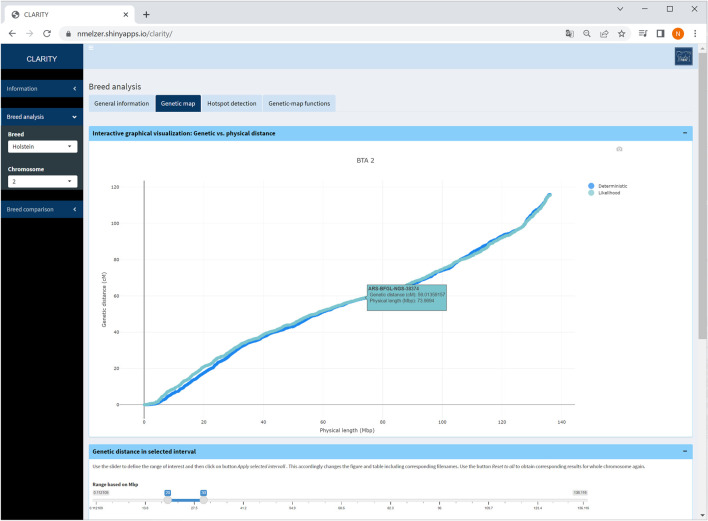
Screenshot of genetic map obtained with the likelihood-based and the deterministic approach on selected chromosome 2 in Holstein cattle. A table below (not shown) contains detailed information on each marker.

Physical and genetic map coordinates are listed for all markers in table or graphical format. If a specific chromosome is selected on the sidebar menu, the user can zoom into relevant chromosomal regions (Figure 4). In each graphic, the genetic-map coordinates of the deterministic and likelihood-based approach appear. The graphical as well as the table output is adaptable to a user specified chromosome window. Selecting the option “all chromosomes” provides a static overview of 29 single graphics.

#### 4.2.3 Hotspot detection

**FIGURE 5:**
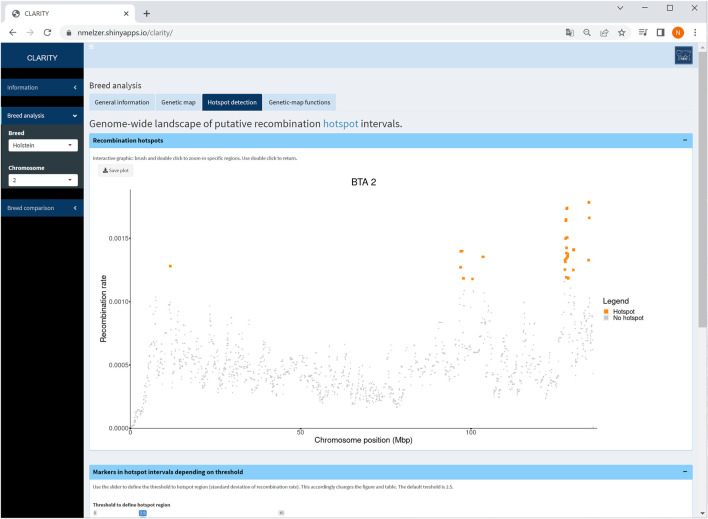
Screenshot of recombination rate between adjacent markers on selected chromosome 2 in Holstein cattle. The coloured points are indicative for putative recombination hotspot intervals using the default threshold. The table below (not shown) provides detailed information on each marker in a hotspot interval.

The CLARITY app offers a landscape of putative recombination hotspots, in which marker intervals with an elevated recombination rate are colour-marked across the bovine genome or a selected chromosome (Figure 5). The default threshold for the recombination rate is adopted from Ma et al. (2015) who defined a hotspot region with a recombination rate exceeding 2.5 standard deviations from the genome-wide average. The threshold is adjustable by the user. Changing the threshold accordingly affects the interactive graphic as well as the corresponding table listing all markers within the hotspot intervals.

#### 4.2.4 Genetic-map functions

**FIGURE 6:**
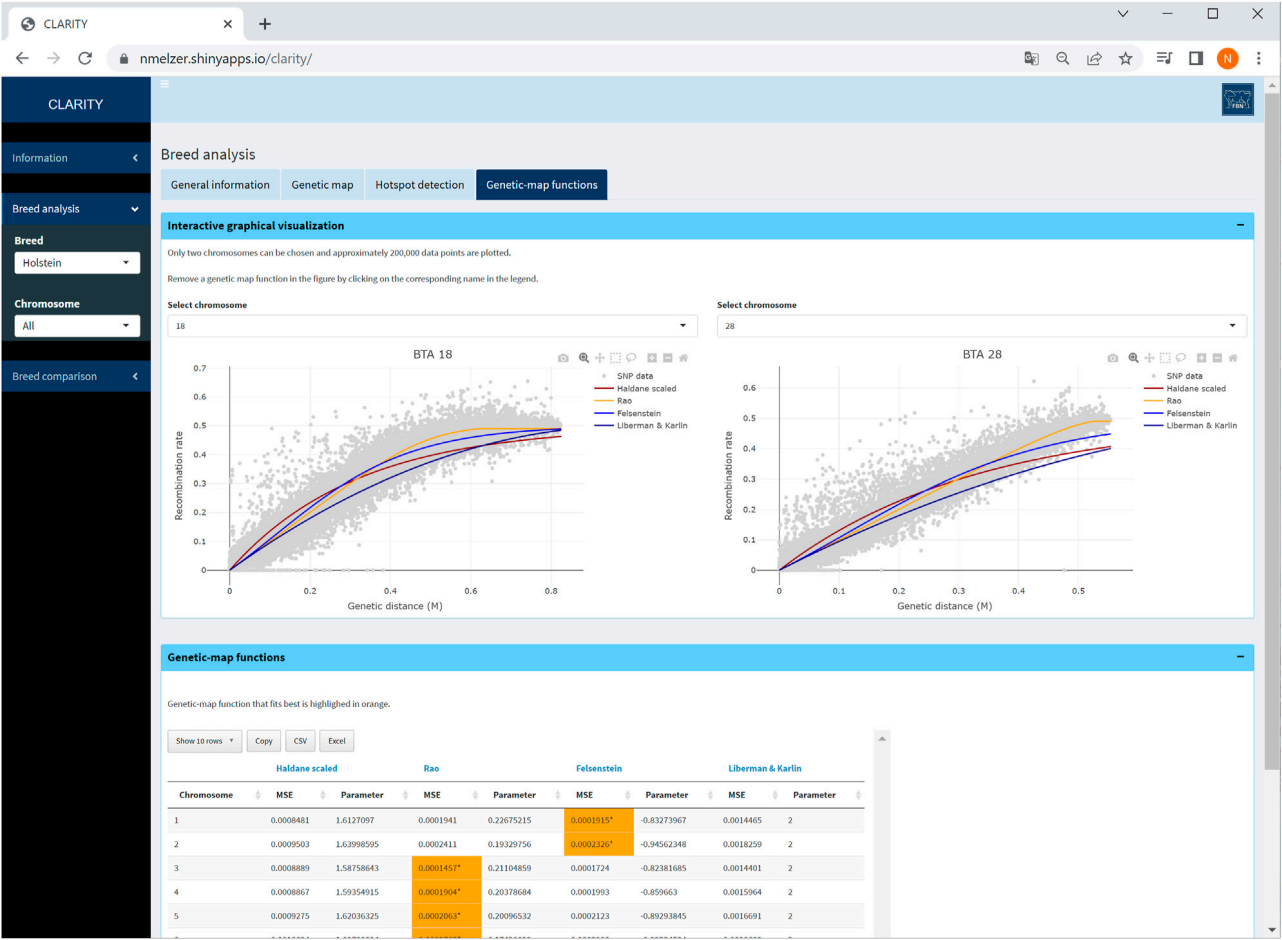
Screenshot of genetic-map functions and its overall fit on two selected chromosomes in Holstein cattle. The table provides details on estimated parameters of genetic-map functions and mean squared error of fitted curves.

The user can investigate the suitability of frequently used genetic-map functions and their overall and local fit to the observed recombination activity (Figure 6). The parameter specifying a genetic-map function was estimated in Step 3 which took all intra-chromosomal marker pairs into account.

The fitted genetic-map functions are illustrated together with a reduced scatterplot of recombination rate *versus* genetic distance for computational reasons. Especially for chromosomes with p≥ 1,415 (i.e., >1,000,000 marker pairs), the computing time is drastically increased retarding the visualisation. To ensure smooth processing, data were thinned based on the Euclidian distance among consecutive data points; a data point is a pair of recombination rate and genetic coordinate ordered according to a vectorised triangular matrix of SNP identifiers. In total, 200,000 data points with largest Euclidian distance were kept. This reduction of data did not impair the visual appearance of the scatter plot.

### 4.3 Breed comparison

The third sidebar menu “Breed comparison” contains comparative analyses between breeds separated into the same tabs as described above. In addition, a Venn diagram summarises numbers of breed-specific and shared SNPs on a selected chromosome or over the entire autosome as well as in hotspot intervals. In the tabs “genetic map” and “hotspot detection”, the Venn diagram is interactively linked with the corresponding table—this allows the user to retrieve particular information of a selected Venn set. The Venn diagram also dynamically adapts to a user defined range and threshold, respectively. Furthermore, since the fit of genetic-map functions might differ between breeds and chromosomes, a barplot displays counts of “best” genetic-map functions in each breed if the option “all chromosomes” is chosen.

**FIGURE 7:**
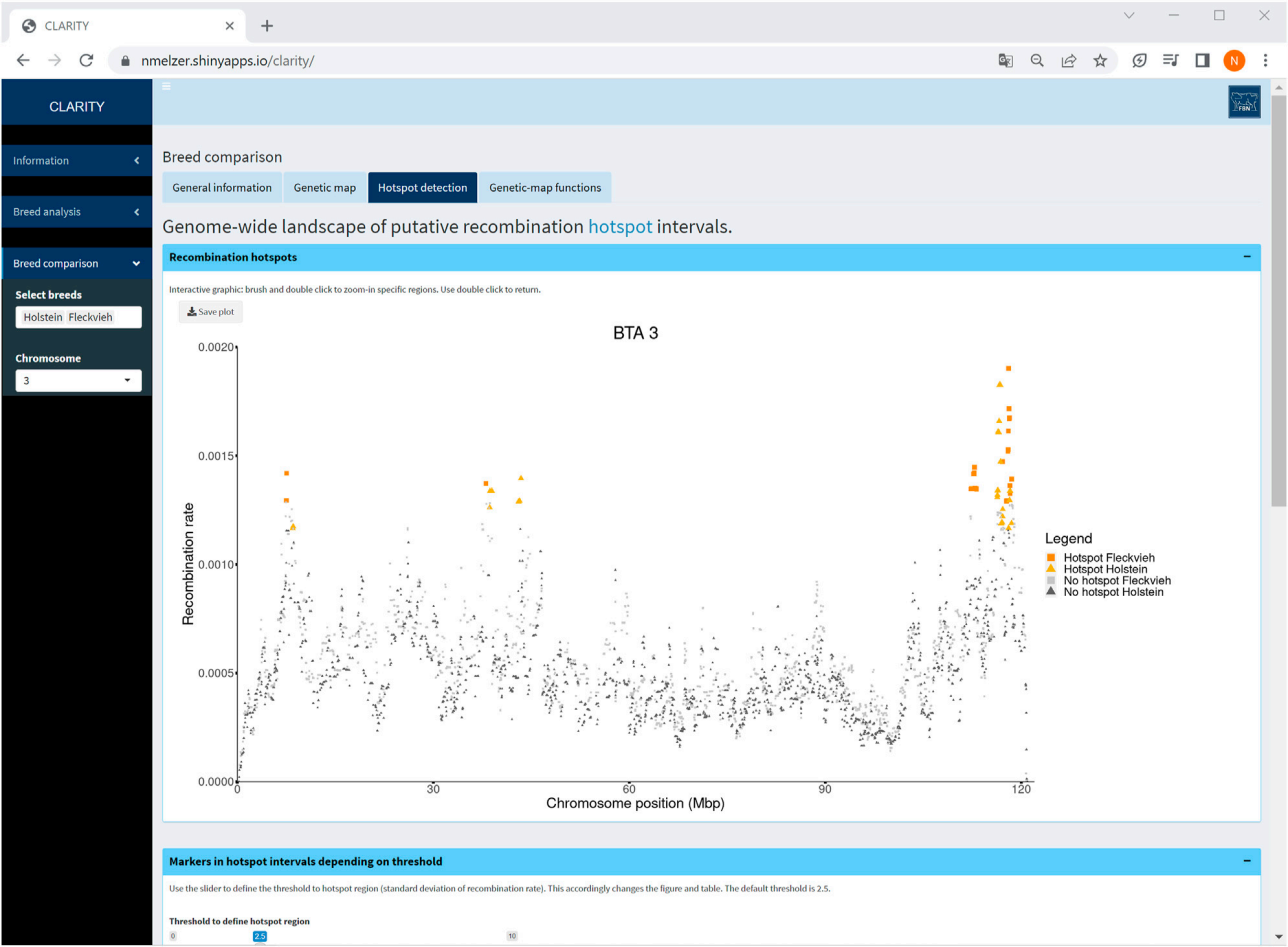
Screenshot of recombination rate between adjacent markers on selected chromosome 3 in a comparison of Holstein and Fleckvieh cattle. The yellow triangles and orange rectangles highlight markers in hotspot intervals of Holstein and Fleckvieh, respectively.

Particularly, a comparison of Holstein and Fleckvieh cattle suggested similar recombination activity genome-wide. As an example, an inspection of hotspot intervals on chromosome 3 (Figure 7) underlined regions of increased recombination rates at the chromosome ends that coincided well in both breeds. Furthermore, for each chromosome, genetic-map functions were almost overlapping. Small deviations of genetic-map functions were observed on chromosomes 5, 6, 9, 15, 16, and 18 with a slightly steeper curve in Fleckvieh.

## 5 Discussion and outlook

The CLARITY app provides an environment to interactively explore the physical and genetic map in selected cattle breeds. Importantly, processing of genotype data and presenting results via Shiny app rely on the current bovine genome assembly ARS-UCD1.2. In case of new assembly releases, Steps 1–3 need to be re-run and an app update becomes necessary. (Especially, both approaches in Step 2 depend on the ordering of markers for inferring phases of sire genotypes and recombination events.) Though a pipeline for Steps 1–3 is available at github, and it could theoretically be part of the Shiny app, we do not recommend its inclusion for computational matters as mentioned in Section 2.

Further work on the integration of data from other breeds (beef, dairy, dual-purpose) is underway and will facilitate complex comparative analyses of map features (e.g., hotspot intervals and assembly flaws) in different genomes. Our Shiny app will be extended accordingly, certainly increasing its value for educational and research purposes.

## Data Availability

CLARITY is a publicly available Shiny app that can be accessed via web interface at https://nmelzer.shinyapps.io/clarity. The corresponding R package CLARITY v1.0.1 including the source code and processed data can be downloaded from https://github.com/nmelzer/CLARITY under the terms of GPL (≥ 2.0). A pipeline for processing genotype data and an R script for composing the app input data are available at https://github.com/wittenburg/hsrecombi. Restrictions apply to the availability of the original data supporting the findings of this study due to thirdparty ownership. Genotype data are available from the Association for Bioeconomy Research (FBF, Bonn) and ZuchtData (Vienna) upon agreement. Requests to access the original datasets should be directed to www.fbf-forschung.de/kontakt.html; www.rinderzucht.at/zuchtdata/team.html.
